# Leukocyte Mitochondrial DNA Copy Number Is Associated with Chronic Obstructive Pulmonary Disease

**DOI:** 10.1371/journal.pone.0138716

**Published:** 2015-09-22

**Authors:** Shih-Feng Liu, Ho-Chang Kuo, Ching-Wan Tseng, Hung-Tu Huang, Yung-Che Chen, Chia-Cheng Tseng, Meng-Chih Lin

**Affiliations:** 1 Division of Pulmonary & Critical Care Medicine, Department of Internal Medicine, Kaohsiung Chang Gung Memorial Hospital, Kaohsiung, Taiwan; 2 Department of Respiratory Therapy, Kaohsiung Chang Gung Memorial Hospital, Kaohsiung, Taiwan; 3 Chang Gung University College of Medicine, Kaohsiung, Taiwan; 4 Department of Pediatrics, Kaohsiung Chang Gung Memorial Hospital, Kaohsiung, Taiwan; 5 Department of Anatomy, School of Medicine, College of Medicine, Kaohsiung Medical University, Kaohsiung, Taiwan; Georgia Regents University, UNITED STATES

## Abstract

**Background:**

Oxidative stress is known to be involved in the pathogenesis of chronic obstructive pulmonary disease (COPD). Evidence suggests that leukocytes mitochondria DNA (mtDNA) is susceptible to undergo mutations, insertions, or depletion in response to reactive oxidative stress (ROS). We hypothesize that mtDNA copy number is associated with the development of COPD.

**Methodology/Principal Findings:**

Relative mtDNA copy number was measured by a quantitative real-time PCR assay using DNA extracted from peripheral leukocytes. MtDNA copy number of peripheral leukocytes in the COPD group (n = 86) is significantly decreased compared with non-smoker group (n = 77) (250.3± 21.5 VS. 464.2± 49.9, P<0.001). MtDNA copy number in the COPD group was less than that in the healthy smoking group, but P value nearly achieved significance (250.3± 21.5 VS. 404.0± 76.7, P = 0.08) MtDNA copy number has no significance with age, gender, body mass index, current smoking, and pack-years in COPD group, healthy smoker group and no smoker group, respectively. Serum glutathione level in the COPD group is significantly decreased compared with healthy smoker and non-smoker groups (4.5± 1.3 VS. 6.2± 1.9 and 4.5± 1.3 VS. 7.1±1.1 mU/mL; P<0.001 respectively). Pearson correlation test shows a significant liner correlation between mtDNA copy number and serum glutathione level (R = 0.2, P = 0.009).

**Conclusions/Significance:**

COPD is associated with decreased leukocyte mtDNA copy number and serum glutathione. COPD is a regulatory disorder of leukocytes mitochondria. However, further studies are needed to determine the real mechanisms about the gene and the function of mitochondria.

## Introduction

Chronic obstructive pulmonary disease (COPD) is a slowly progressive condition characterized by poorly reversible airflow limitation that is usually progressive and associated with an abnormal inflammatory response of the lung. Smoking has been implicated as the main etiological factor for the development of COPD [1]. The sources of the increased oxidative stress in the respiratory compartment in patients with COPD derive from the increased burden of oxidants in cigarette smoke, and from the increased amounts of reactive oxygen and nitrogen species (ROS and RNS) released from leukocytes and macrophages involved in the inflammatory process in COPD [2]. Epidemiological findings during the last decade have also indicated potential protective effects of antioxidant vitamins in the development and clinical course of COPD [3]. Increasing evidence suggests oxidative stress and inflammation in the lungs may also be responsible for many of the systemic effects of COPD [4]. Markers of oxidative stress have also been demonstrated in different bodily compartments. The co-morbidities in COPD are associated with a result of 'overspill' of inflammatory mediators from the lungs [5]. Muscle wasting and coronary artery disease are highly related to the morbidity and mortality in these patients and have even been found to be present in a considerable proportion even in mild COPD [6]. In addition, peripheral blood leukocytes in COPD patients have been shown to release more ROS than in normal subjects, which may contribute to morbidity and mortality [7].

Mitochondria are the eukaryotic organelles responsible for energy production through the synthesis of ATP. In normal cells, mitochondria have 2–10 copies of their genomes [8]. Mitochondria DNA (mtDNA) is a circular molecule that lacks introns and protective histones. As a consequence, the mutation rate for mtDNA is substantially greater than that of nuclear genomic DNA. Further, mitochondria have limited DNA repair capacity and compensate for damage by increasing the number of mtDNA copies [8]. MtDNA in leukocytes in COPD patients may easily undergo mutations, insertions, or depletions in response to oxidative stress. A previous study have proved that mitochondrial changes in COPD epithelium are potentially the consequence of long-term exposure to cigarette smoke, leading to impaired mitochondrial function and may play a role in the pathogenesis of COPD [9]. Meyer et al also have demonstrated that the vastus lateralis muscle in COPD patients presented with alterations that include a decrease in mitochondrial density and biogenesis, impaired mitochondrial respiration and coupling, as well as increased mitochondrial production of reactive oxygen species, possibly associated with increased mitochondrial apoptosis [10]. Exercise may enhance the decrease in mtDNA content of skeletal muscle probably due to oxidative stress in COPD patients [11]. In addition, Kim et al demonstrated that peripheral leukocyte mtDNA copy number was positively correlated with leukocyte telomere length in community-dwelling elderly women. Their findings suggest that telomere function may influence mitochondrial function in humans. [12]. COPD patients have short leukocyte telomeres, which are in turn associated increased risk of total and cancer mortality [13].

Given that mitochondria are highly susceptible to ROS [14], mtDNA copy number may serve as a biomarker for exogenous and endogenous exposures that are associated with subsequent tobacco-related COPD risk. We hypothesized that mtDNA copy number of peripheral leukocytes may be associated with the development of COPD.

## Materials and Methods

### Ethics Statement

The study was approved by Chang Gung Memorial Hospital Research Ethics Committee (IRB#99-0890B), which was obtained before the study began. Written informed consents were obtained from all participants. The written informed consent had fixed format which included the study topic, brief introduction, purpose, test methods and procedures: inclusion / exclusion criteria, possible side effects and dangers of the study, expected study results; other possible methods of treatment and description, handling of emergency situations, remaining specimen charge, participant interests, and the related signatures and date including participant, participant medical record number, legislative consent people and the relationship with participant, phone number, witnesses, and study moderator. Before the study began, study proposal and written informed consent were checked by the Hospital Research Ethics Committee until they were considered appropriate after revisions. The study moderator explained the contents according to the informed consent form to the participant in detail. If the participant agreed to accept the test as witnessed, the participant himself or his legal representative signed the consent form.

### Design and Study Participants

All candidates were recruited from the outpatient clinic of the Department of Pulmonary Medicine and the Department of Health screening center, Kaohsiung Chang Gung Memorial Hospital, Taiwan. We collected three groups including COPD patients, healthy smokers and non –smokers. Oxidative stress is known to be involved in the pathogenesis of COPD. MtDNA copy number of peripheral leukocytes and serum glutathione level were analyzed in the three groups. Inclusion criteria for entry are as follows: All COPD patients with age more than 40 years and at least a 10 pack per year smoking history have forced expiry volume in 1 s (FEV_1_)/forced vital capacity (FVC) ratio <0.7, and FEV_1_ percent predicted <70% reversibility with inhaled bronchodilators of <15% of predicted FEV_1_. Stable COPD patients were defined as those using bronchodilators but not inhaled or oral corticosteroids, and had no exacerbation in the previous 6 weeks. For COPD subjects, chest radiographs and histories were evaluated to exclude other causes of airflow limitation such as pulmonary tuberculosis, bronchial asthma, bronchiectasis, and heart failure. Control groups including healthy non- smokers and healthy smokers with at least a 10 pack per year smoking history were enrolled based on history, physical examination, and spirometric data with FEV1/FVC ratio >70% and FEV_1_, >80% predicted. In addition, for prevention of mtDNA copy number influenced by other associated diseases, all candidates with renal cell carcinoma, liver disease, biliary atresia, cardiomyopathy, breast cancer, or end renal stage disease were excluded.

### DNA purification from peripheral whole blood

The steps of DNA purification from peripheral whole blood are described as below: 3ml whole blood is centrifuged 3000rpm for10min, taking about 250 μl Buffy coat (QIAGEN, Germany) added 900ul RBC Lysis Solution which is shaken up and down for several times, to observe if the solution gradually turns red hyaline, then centrifuged at 2000rpm for10min until bottom of the tube appear white pellet, and discard the upper supernatant. To add 500ul Cell Lysis Solution and vortex for several minutes until the cell pellet is completely dissolved away. Sample placed on ice for about five minutes, then add 130ul Protein Precipitation Solution and vortex for a few seconds, then placed on ice for a few minutes, centrifuged 12,000 rpm for 10 min at 4°C, the protein pellet will become tight. Taking the supernatant to another tube new eppendorf and add 300ul isopropanol, mixing, shaking up and down several times, there will be filaments, centrifuged 12000 rpm for 10 min at 4°C, the supernatant is discarded, leaving the pellet. Adding 500 ul 70% ethanol to wash pellet, and then centrifuged at 12000 rpm for 10 min at 4°C. The supernatant is discarded, and leave the pellet dry. Once completely dry, add about 50ul DNA Hydration Solution, place 65°C for 1h to dissolved cell pellet and complete DNA extraction. The DNA is stored at -80°C.

### MtDNA copy number of peripheral white blood cell

The mtDNA content was determined using a *7900HT Sequence Detection Systems* (Applied Biosystems) in duplicate. PCR primers for “specific gene” were designed using Primer Express V.2.0 software (Applied Biosystems Inc., Foster City, CA) based on the specific sequences from GenBank. The mitochondrial cytochrome b gene forward primers were 5- CATCATTGGACAAGTAGCATCCGTAC -3, and the reverse primer was 5- TTTCATCTCCGGTTTACAAGACTG -3. The mtDNA content was corrected by simultaneously measuring nuclear DNA. The RNase P gene is a single-copy gene that encodes the RNA moiety for the RNase P enzyme. The RNase P gene forward primers were 5- AGCCATCTTGAGAATATGTAGCAGG -3, and the reverse primer was 5- ATTTTTGCTCCATCGGTCTCTC -3. The thermal cycling conditions for real-time PCR were 50°C for 2 mins, then 95°C for 10 mins, and 40 cycles of denature (95°C, 15 secs) and annealing/extension (60°C, 60 secs). After PCR cycles, dissociation curve examination was performed. The amount of mtDNA was expressed relative to the quantity of RNAse P (2^(-ΔCT)^) and in terms of ΔCT values calculated by using sequence detector software version 2.3. A second area of the genome was selected to confirm the accuracy of the results (S1 File).

### Measurement of serum glutathione level

The levels of serum glutathione were also enzymatically assessed with glutathione reductase using a commercial glutathione assay kit (Cayman Chemical, Ann Arbor, MI, USA) in accordance with the manufacturer’s instructions. In brief, add 50ul of Standard and sample to each well, and cover the plate with the plate cover provided, and prepare the Assay Cocktail by mixing the following reagents in a 20ml vial: MES Buffer(11.25ml),reconstituted Cofactor Mixture (0.45ml), reconstituted Enzyme Mixture (2.1ml), water (2.3ml), and reconstituted DTNB(0.45ml), then remove the plate cover and add 150ul of the freshly prepared Assay Cocktail to each of the wells containing standards and samples. Replace the plate cover and incubate the plate in the dark on an orbital shaker and measure the absorbance in the wells at 405 nm using a plate reader.

### Lung Function Testing

Lung function was evaluated with a Sensor Medics Vmax Spectra Series, model 29 series (Yorba Linda, CA92887). Each measurement was performed 15 min after the inhalation of 400 μg of salbutamol via a metered-dose inhaler.

### Statistical analysis

11.5 SPSS was used to perform all statistical tests. Values are represented as mean±SD. Student's t-test with two tailed values was performed to compare the differences between the two groups. Multiple comparisons among three groups were analyzed by one-way analysis of variance followed by post Hoc corrections with tukey method. Correlation between two continuous variables was made using Pearson correlation test. P<0.05 was considered statistically significant.

## Results

### Patient Characteristics

The baseline characteristics of COPD group and controls are listed in Table 1. Eighty-six COPD patients, 33 healthy smokers and 77 non-smokers are enrolled in this study. The variables include age, gender, body mass index, lung function, current smoking, pack-years, mtDNA copy number, and serum glutathione level. Age, gender, lung function, current smoking, pack-years, mtDNA copy number, and serum glutathione level show significant among three groups (P<0.05) and the significance between groups also present in Table 1. Body mass index show no significance among three groups (Table 1). Means of relative mitochondria DNA copy number of peripheral leukocytes show no significance with age, gender, current smoking, and pack-years in COPD group, healthy smoker group and no smoker group respectively (Table 2).

**Table 1:** Baseline characteristics of COPD group, healthy smoker group and no smokers group.

	COPD (n = 86)	Healthy smoker (n = 33)	No smoker (n = 77)	P value
Age	69.1± 11.2	56.1± 10.0*	54.8± 0.6*	<0.001
Gender (male %)	95	97	83*	<0.017
FEV1/FVC (%)	52.9± 11.7	80.2± 6.3*	86.7 ± 7.4* ^#^	<0.001
FVC (%)	81.1 ± 20.3	88.9 ± 13.3	90.1 ± 10.9*	0.04
FEV1 (%)	56.6 ± 20.6	89.6 ±15.7*	95.8 ± 10.4*	<0.001
Pack-years	51.2± 40.0	34.9± 20.7*	0	0.004
Current smoking (%)	32.1	57.6*	0* ^#^	<0.001
BMI (kg/m^2^)	23.6± 4.2	24.6 ± 3.5	24.1± 2.9	0.353
MitDNA Copy number	250.3± 21.5	404.0± 76.7	464.2± 49.9*	<0.001
Glutathione (mU/mL)	4.5± 1.3	6.2± 1.9*	7.1±1.1* ^#^	<0.001

*vs. COPD group, P<0.05;

^#^vs. healthy group, P<0.05

MitDNA-mitochondria DNA; BMI: Body mass index

**Table 2:** Relative mitochondria DNA copy number related to variables in COPD group, healthy smoker group and no smoker group respectively.

Variable	COPD group	healthy smoker group	No smoker group
Age	P = 0.92	P = 0.42	P = 0.35
Gender	P = 0.85	P = 0.58	P = 0.47
BMI	P = 0.90	P = 0.29	P = 0.47
Pack-years	P = 0.13	P = 0.34	---
Current smoking	P = 0.41	P = 0.30	P = 0.91

BMI: Body mass index

### MtDNA Copy Number of Peripheral Leukocytes is Associated with Chronic Obstructive Pulmonary Disease

**Fig 1:**
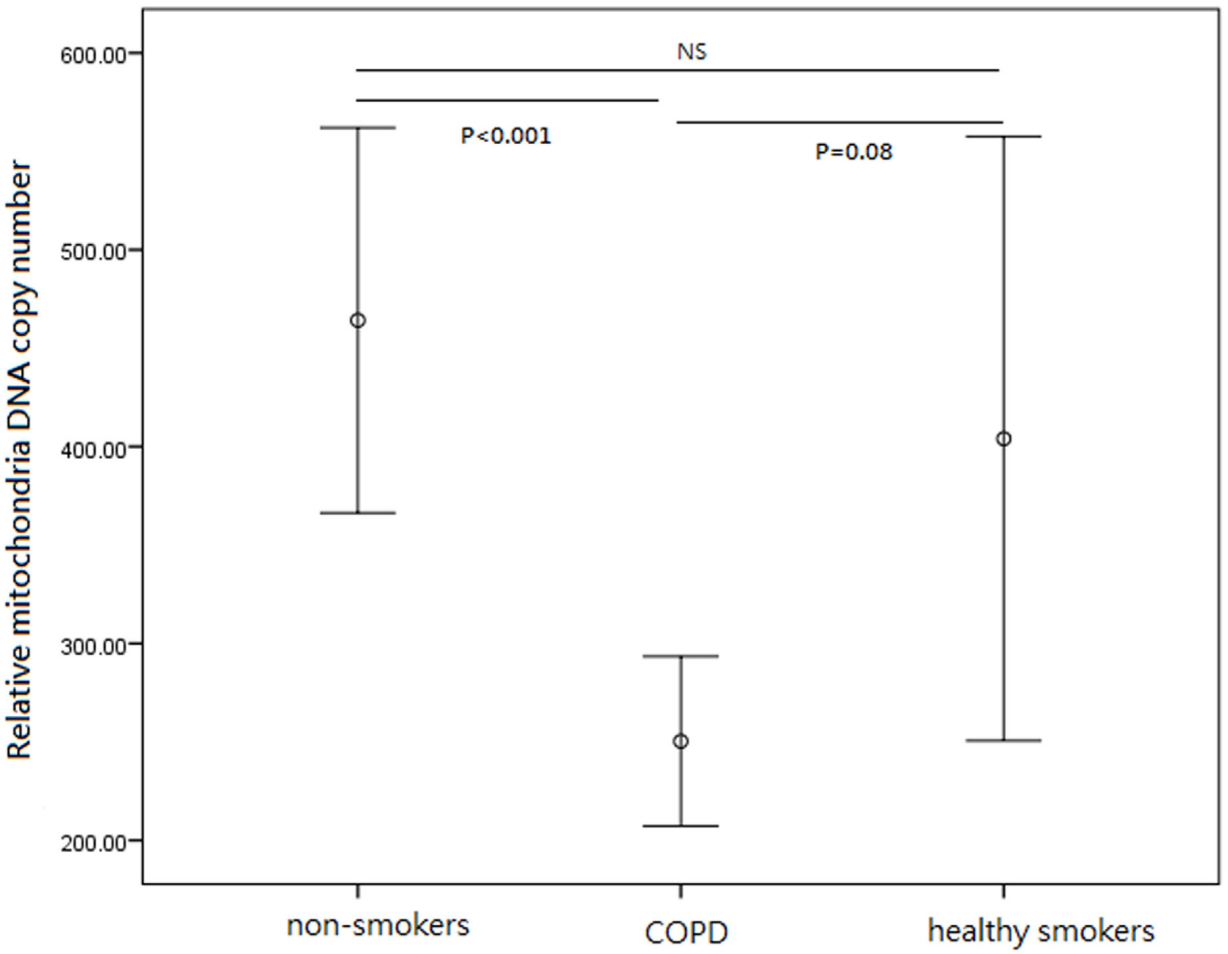
Relative mtDNA copy number of peripheral leukocytes in COPD group, heathy smokers and no-smokers. MtDNA copy number: COPD group (N = 86, 250.3± 21.5); heathy smokers group (N = 33, 404.0± 76.7); non-smokers group(N = 77, 464.2± 49.9). Results are expressed as mean ± standard error. P<0.001, COPD group vs. non-smokers group; P = 0.08, COPD group vs. heathy smokers group; NS, non-smokers group vs. heathy smokers group.

MtDNA copy number of peripheral leukocytes in the COPD group was significantly decreased compared with no-smoker group (250.3± 21.5 VS. 464.2± 49.9, P<0.001) (Table 1 and Fig 1). MtDNA copy number of peripheral leukocytes in the COPD group was less than that in the healthy smoker group, but P value nearly achieved significance (250.3± 21.5 VS. 404.0± 76.7, P = 0.08). MtDNA copy number of peripheral leukocytes is similar in the healthy smoker and no-smoker groups (404.0± 76.7 VS. 464.2± 49.9, P = 0.69).

### The Relationship between Serum Glutathione Level and mtDNA Copy Number of Peripheral Leukocytes/ Chronic Obstructive Pulmonary Disease

**Fig 2:**
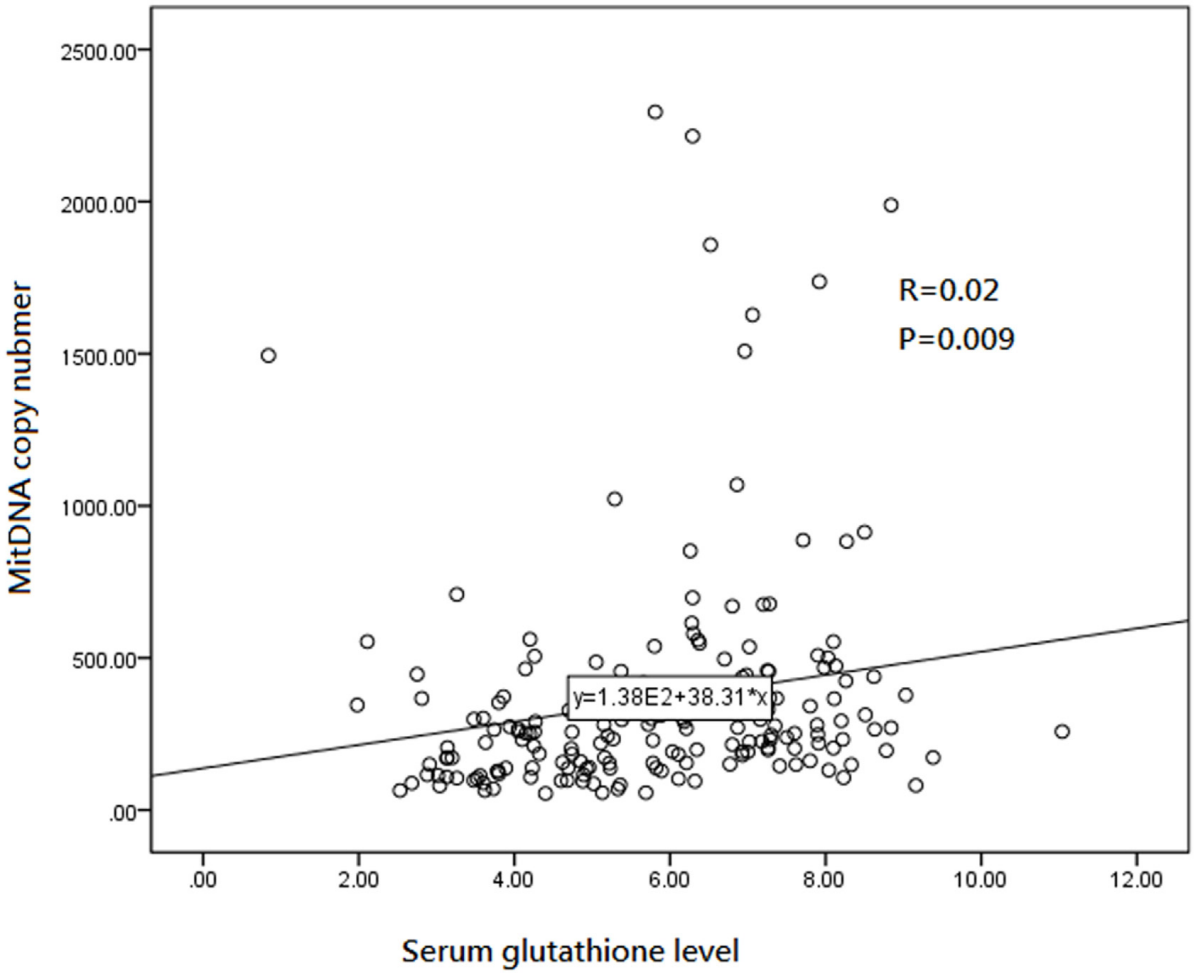
Relative mtDNA copy number of peripheral leukocytes correlates with serum glutathione level. Pearson correlation test shows a significant liner correlation between mtDNA copy number and serum glutathione level (R = 0.2, P = 0.009).

Serum glutathione level in the COPD group is significantly decreased compared with healthy smoker and non-smoker groups (4.5± 1.3 VS. 6.2± 1.9 and 4.5± 1.3 VS. 7.1±1.1 mU/mL; P<0.001 respectively) (Table 1). Serum glutathione level in the healthy smoker group is significantly decreased compared with non-smoker group (6.2± 1.9 VS. 7.1±1.1 mU/mL, P = 0.04). MtDNA copy number of peripheral leukocytes has significant liner correlation with serum glutathione level (R = 0.2, P = 0.009) (Fig 2).

## Discussion

This study results show that COPD is associated with decreased peripheral leukocytes mtDNA copy number of which reveals COPD is a regulatory disorder of dysfunctional mitochondria by ROS in peripheral leukocytes. Smoking is an important risk factor for COPD. However, in all smokers, about 15–20% of these smokers will become COPD. Someone with persistent smoking in their lives will not become COPD, thus we know that the gene plays a very important role in the development of COPD [15]. Although we do not know the real relationship between nuclear DNA and MtDNA, this concept is consistent with our results that show mtDNA copy number in COPD group is significantly lower than for non-smokers. MtDNA copy number is similar in healthy smoker and non-smoker groups. The COPD group, which is susceptible to the COPD gene, has less mtDNA protection or biosynthesis than non-smokers. In contrast, healthy smokers without susceptibility to the COPD gene may have protective effect on mtDNA destructed by ROS, thus their mtDNA copy number is similar to that in non-smokers. We infer that nuclear DNA plays a critical role in mitochondrial biogenesis or protection. This is compatible with the previous findings that lung epithelium from COPD patients is more prone to cigarette smoking-induced mitochondrial damage/dysfunction and premature ageing, which may eventually affect lung function. In contrast, non-susceptible to develop COPD individuals have better protection against cigarette smoke induced changes in mitochondrial function [9]. Future studies will be of interest to assess the gene and the function of mitochondria differs between individuals who are non-susceptible and susceptible to develop COPD.

Apoptosis can be physiological but can also lead to disease, including muscle atrophy [16]. Mitochondria are central actors in the intrinsic apoptotic pathway. While, an extrinsic pathway independent from the mitochondria can also trigger apoptosis through the involvement of death receptors, such as Fas and tumour necrosis factor receptor-1 [10]. Evidence has demonstrated that the extrinsic apoptotic pathway is important in muscles of COPD patients [17]. Electron microscopy has also demonstrated a reduction in mitochondrial number and fractional area in the vastus lateralis in COPD patients in comparison to control subjects [18]. Furthermore, evidences demonstrate that involvement of the peroxisome proliferator-activated receptors (PPARs) and PPAR-γ coactivator (PGC)-1α in regulation of skeletal muscle morphology and metabolism, and mitochondrial transcription factor A (TFAM) has been implicated in the process of mitochondrial biogenesis [19]. TFAM protein is significantly lower in the quadriceps muscle of patients with moderate-to-very severe COPD than in controls and TFAM mRNA and protein are also significantly lower in patients with cachectic COPD compared to that in both non-cachectic patients and control subjects [19] The level of TFAM protein is related to apoptosis of pulmonary vascular endothelial cells. Aberrant TFAM methylation may also play an important role in the pathogenesis of COPD [20]. Consequently, a decrease in the activity of mitochondrial oxidative enzymes has strong relations to muscle performance of COPD patient [21, 22].

Cigarette smoke generates intracellular ROS production of which mitochondria are the main producers. ROS can damage important cellular components like nuclear DNA and organelles. Mitochondria can protect themselves and the cell from oxidative damage in several ways, such as by producing anti-oxidant scavengers, regulating the oxidative phosphorylation (OXPHOS) process responsible for ATP generation, exchanging mtDNA through fission/fusion proteins and PTEN induced putative kinase 1 (PINK1) [23–25] and peroxisome proliferatoractivated receptor gamma co-activator 1-alfa (PPARGC1α) to control mitochondrial biogenesis [26, 27]. Excessive oxidative stress and/or an imbalance or depletion of key mitochondrial fission and fusion markers, including Dynamin-related protein 1 (Drp1), Mitochondrial fission 1 protein (Fis1), Mitofussion (Mfn1 and Mfn2), Optic Atrophy 1 (OPA1) and mitochondrial transcription factor A (Tfam) can lead to mitochondrial damage and disorganized and aberrant cristae formation [28]. Furthermore, it will augment ROS production and cellular apoptosis through cytochrome-C release [29, 30]. Upon persistent mitochondrial damage or oxidative stress exceeding the anti-oxidant response, mitochondrial dysfunction will be introduced. Mitochondrial dysfunction and ROS from cigarette smoke may eventually induce inflammation, and the onset of COPD

The maintenance of intracellular redox homeostasis is dependent on a complex web of antioxidant molecules. Glutathione are important low molecular weight molecules antioxidant presenting in millimolar concentrations within cells. GSH levels of the vastus lateralis were decreased in COPD patients, which was associated with reduced glutamate concentrations. Engelen et al. found that glutathione metabolism was impaired in COPD [31]. The regulation of antioxidant levels in cells is intimately tied to the levels of intracellular ROS and sources of oxidant production. The Nrf2 transcription factor that regulates the expression of a host of antioxidant and detoxifying genes by binding to promoter sequences containing a consensus antioxidant response element [32]. In mammals, mitochondrial biogenesis, the physiological induction of new mitochondria, is regulated by the transcriptional coactivator PGC-1. Interestingly, PGC-1 appears to regulate new mitochondrial formation while simultaneously regulating antioxidant expression [33]. This coordination of new ROS-producing organelles with increased antioxidant levels presumably helps maintain redox homeostasis The current results on the reduction of antioxidant capacity in COPD patients, as suggested by reduced serum levels of glutathione, indicates persistent mitochondrial damage or oxidative stress exceeding the anti-oxidant capacity and leads to leukocytes mitochondrial dysfunction. Subsequently, mitochondrial dysfunction induces inflammation, and the onset of COPD. High-dose treatment with *N*-acetylcysteine has been shown to prevent exercise-induced oxidative stress and significantly improve quadriceps endurance [34]. Antioxidant treatment may have potentially innovative management for mitochondrial pathophysiology in leukocytes dysfunction associated with COPD.

Abnormal peripheral leukocytes in COPD patients due to mitochondrial dysfunction will result in abnormal functions, including shape, expression, differentiation, chemotaxis, and migration. These abnormal functions of leukocytes will involve the pathophysiology of COPD. Yamagata et al. have demonstrated that both CD-11b and CXCR1 expression of peripheral leukocytes are significantly higher in COPD patients than in healthy smokers and the expression of CD-11b and CXCR1has a significant negative correlation with the severity of airflow limitation [35]. Mitochondrial pathophysiology represents an emerging area of research in leukocytes dysfunction associated with COPD and has promising therapeutic implications.

### Limitation of the study

This study has some important limitations that should be kept in mind when interpreting the results. First, the cross-sectional nature of the study design can only explain decreased peripheral leukocytes mitochondria dysfunction is associated with COPD. Although MitDNA copy number is not correlated to FEV1 or FEV1/FVC in COPD group in this study. It needs a longitudinal study to confirm the relationship between severity and prognosis of COPD with MitDNA copy number. Second, several attempts have been made to describe how the numbers of mitochondria correlate with age, although with inconclusive results. In our study, age is not related to leukocyte MitDNA copy number in COPD and control groups. However, age matched control study will make the results indeed completely free from interference by age.

### Conclusion

COPD is associated with decreased leukocyte mtDNA copy number and serum glutathione. COPD is a regulatory disorder of leukocytes mitochondria. However, further studies are needed to determine the real mechanisms about the gene and the function of mitochondria

## Supporting Information

S1 File Relative copy number in another 2 areas of Human CYTB gene in case / control groups. Another 2 areas of CYTB gene were selected to confirm the accuracy of the data, and the results indicate the new data and the previous data with the same trend.
(XLSX)
